# Quality criteria for evaluating creative and interactive science dissemination in public outreach efforts: A scoping review

**DOI:** 10.1371/journal.pone.0328800

**Published:** 2025-07-24

**Authors:** Priscilla Van Even, Bieke Zaman, Karin Hannes

**Affiliations:** 1 Meaningful Interactions Lab, Faculty of Social Sciences, KU Leuven, Leuven, Belgium; 2 Research Group TRANSFORM, Faculty of Social Sciences, KU Leuven, Leuven, Belgium; University of Alberta, CANADA

## Abstract

Creative and interactive science dissemination is gaining recognition as an important component of public science communication, particularly in efforts to bridge the gap between science and society. This scoping review aims to identify science-based quality criteria to develop a conceptual framework for evaluating the quality of public dissemination practices. The research question of this review is: What criteria are described and used in the literature to assess the quality of creative and interactive public science dissemination practices? We mapped, identified, and synthesized key criteria by conducting a scoping review in accordance with the Joanna Briggs Institute (JBI) guidelines. The review focused on studies from 2004 onwards, reflecting the growing role of digital platforms and the rise of social media in public science dissemination. An initial search retrieved 8745 studies from the ERIC (USDE), Web of Science (Clarivate), Scopus (Elsevier), ProQuest Central (ProQuest), Open Dissertations (EBSCO), and Communication and Mass Media Complete (EBSCO) databases. After a three-stage screening process by multiple, independent screeners, 18 studies were selected that met the inclusion criteria (perspective, outcome, phenomenon of interest, and study domain). The framework was developed through iterative thematic analysis. Its comprehensiveness, practical relevance, and applicability were further examined through face validity workshops with practitioners and academics. The resulting evaluation framework comprises three thematic sets of criteria: normative criteria (addressing the scientific base of the dissemination), substantive criteria (covering translation, presentation, and design of the science dissemination), and performative criteria (focusing on the impact of the dissemination). This framework is intended to support the development of science communication that is engaging, inclusive, and impactful for broad audiences, while also encouraging reflective practice among science communicators.

## 1. Introduction

Creative and interactive science dissemination practices have become an important part of scientific knowledge translation in public outreach efforts to bridge the gap between science and citizens. In this study, we refer to science dissemination as a specific form of science communication, focused on the public disclosure of research and project results. We rely on the description provided by the European Commission’s Research & Innovation Participant Portal Glossary [1]. Whereas *science communication* is an umbrella term that encompasses a wide range of *outreach efforts* throughout the entire research trajectory, *science dissemination* is typically positioned as the final step in the whole chain of scientific activities and serves as a step towards scientific knowledge translation and practice change [2,3]. The objective of science dissemination is to translate scientific findings in a way that enables audiences to consider using and applying them [4, p. 195].

The rise of the internet and the proliferation of modern technologies have transformed processes of knowledge translation and science dissemination, as well as citizens’ involvement in science [5–7]. Digital media affect how scientific information is communicated, increasing public access and facilitating worldwide diffusion of the latest knowledge 24/7. What is more, the mediatization of science and ongoing technological developments have given rise to new and creative ways of science outreach. While older practices of science dissemination remain valuable, digital media have transformed the way humans perceive, engage with, and experience scientific knowledge and science outreach practices [7]. The transformative impact of these mediatization processes has ushered in a new era of science outreach, characterized by new, creative, and interactive forms of public engagement with science and its communication.

The use of creative and interactive science dissemination practices helps to make science memorable, meaningful, and accessible for people beyond academia [8,9]. Moreover, creative dissemination forms are likely to positively impact people’s engagement and motivation to communicate research findings, encourage self-reflection, and improve their understanding of theoretical concepts [10–14]. Interactive engagement with science holds strong potential to enhance public involvement and accessibility, both of which are key to effective dissemination, knowledge translation, and narrowing the gap between science and society [3,5,15–17].

These dissemination practice transformations extend beyond science outreach to the scientific enterprise itself. Digital media and technologies have made public science dissemination, and the public engagement with science in general, a fundamental part of how science is practiced in the 21st century. The ‘popularization’, ‘openness’ and ‘democratization’ of science brought by the digital revolution offers citizens opportunities for empowerment and a stronger capacity to use scientific knowledge [5,6]. However, at the same time, these technology-mediated outreach practices also harbor the risk of quality loss in the communication process and scientific misinformation [4, pp.139–140]. Poor, inadequate, or insufficient science dissemination does not only risk to obstruct the public use of scientific information; it can also inflict lasting damage to the trust between scientists and the public [18]. Hence, adequate public science dissemination plays a pivotal role in fostering and mediating trust in science among members of the public [19]. Avoiding or mitigating the risks of inadequate, unsuccessful, or even damaging public science dissemination of research results is thus important to restore or maintain a healthy relationship between citizens and scientists. Science communicators play an important role here. This raises not only the question of how to best safeguard the quality of science translations to citizens but also, and primarily, the question of what can be considered a successful and valuable form of public science dissemination.

Although there is a substantial body of research on evaluation criteria for assessing the scientific quality of *research findings*, a comprehensive literature review of criteria for evaluating the scientific quality of the *dissemination of research findings* remains lacking. Yet, the evaluation of science communication and dissemination practices is essential to safeguard its scientific quality and credibility [20,21]. Olesk et al. (2021) have offered a valuable holistic framework with quality indicators for public science communication, developed through expert workshops [20]. While their study provides important insights into the broader field of science communication, it does not specifically focus on dissemination practices, nor is the framework discussed against the existing body of research. To date, we thus lack a synthesized overview of existing evaluation criteria relevant to public science dissemination. To address this gap, we conducted a scoping review to identify and map out relevant evaluation criteria that have remained scattered throughout the existing literature. Our focus is on evaluation criteria relevant to interactive and creative dissemination practices that are accessible and engaging to the broader public. This focus aligns with the current paradigm change in the field of science communication, which challenges traditional, top-down scientific practices and concepts and promotes a wide range of interactive and creative dissemination practices [4].

This study addresses the following research question: “What criteria are described and used in the literature to assess the quality of creative and interactive science dissemination practices for public outreach?”. It contributes to the scientific literature by identifying and mapping evaluation criteria from existing studies, laying the groundwork for evidence-based guidance on how to critically assess creative and interactive public science dissemination practices. By identifying and mapping these criteria, the study contributes to the critical evaluation of the quality of science dissemination practices, an essential step in safeguarding the quality of science dissemination and communication. Moreover, the findings of our review support the development of science dissemination that is engaging, inclusive, and impactful for broad audiences, while fostering reflective practice among science communicators including researchers, media professionals, museum professionals, and those involved in creative and artistically inspired public outreach events.

## 2. Method

The scoping review was conducted in accordance with the Joanna Briggs Institute (JBI) guidelines and followed four key steps: (1) development of the search strategy, (2) screening and data extraction, (3) synthesis, and (4) expert consultations through face validity checks. The research project began in March 2020 and concluded in January 2025. The documentation of the protocol and framework iterations is available via Zenodo and OSF [22,23].

### 2.1. Search strategy

Databases: We conducted searches in various databases that encompass the domains of education, humanities, social sciences, and interdisciplinary connections to the arts and design. Specifically, we consulted the following databases: ERIC (USDE), Web of Science (Clarivate), Scopus (Elsevier), ProQuest Central (ProQuest), Open Dissertations (EBSCO), Communication and Mass Media Complete (EBSCO).

Sources: We searched both academic and non-academic types of information sources in the databases and included journals, books, encyclopedias, dissertations, expert opinions, and magazine articles, retrieved through a scientific, systematic, and transparent research process.

Time period: We chose 2004 as the starting point for our review because of the significant rise in social media platform usage, marked by a million monthly active users on Myspace in 2004 and the launch of Facebook in the same year, as indicated by the Our World in Data platform [24]. The beginning of the twenty-first century can be considered the start of modern social media, which had a significant impact on the dissemination of artistic, design-related and other forms of creative public outreach initiatives. This temporal limitation does not imply that we exclusively focused on digital forms of public outreach for our criteria list. It rather implies that we have been looking for criteria that align with our contemporary societal and scientific time spirit.

Search terms: An overview of the search terms, as clustered in three search strings, can be found in Table 1. The *first* string focused on the type of perspective and was added to ensure that the information sources collected in the search were specifically related to the dissemination of knowledge that was the result of a scientific endeavor. The *second* string encompassed a wide array of interactive and creative dissemination practices and tools. In the *third* search string, we targeted information sources that presented evaluation criteria or discussed quality assessment or critical appraisal.

**Table 1:** Overview of the search terms and strings.

Terms to capture perspective:
(“scien*” OR “research” OR “knowledge”)
**Terms to capture creative and interactive dissemination:**
((“creativ*” OR “senses” OR “sensorial*” OR “multi-sensorial” OR “artistic”) OR (“arts-based” OR “art-based” OR “arts-informed” OR “art* based” OR “art* informed” OR “media” OR “social media” OR “new media” OR “blog*” OR “video*” OR “broadcast*” OR “television” OR “TV” OR “film*” OR “online channel*” OR “podcast*” OR “radio*” OR recording*” OR “game*” OR “gaming” OR “webinar*” OR “website*” OR “VR” OR “virtual reality” OR “virtual world” OR “AR” OR “augmented reality OR “event*” OR “exhibit*” OR “theat* exhibit*” OR “museum exhibit*” OR “photo-elicitation” OR (visual* W/5 (work* OR form* OR art* OR method* OR practice* OR audio* OR present* OR represent* OR design* OR creativ* OR interact* OR engag* OR output* OR outreach* OR display* OR disseminat* OR communicat*)) OR “photovoice” OR “photo-voice” OR “photograph*” OR “design*” OR “paint*” OR “collage*” OR “cartoon*” OR “comic*” OR “animation*” OR “photocomic*” OR “image*” OR “draw*” OR “infographic*” OR “quilt” OR (perform* W/5 (work* OR form* OR art* OR method* OR represent* OR present* OR practice* OR creativ* OR interact* OR engag* OR output* OR outreach* OR display* OR disseminat* OR communicat*)) OR “danc*” OR “drama*” OR “music*” OR “sound art*” OR “theatre” OR “theater” OR “artwork*” OR “creative art*” OR “literary” OR “poetry” OR “poetic*” OR “poem*” OR “fiction*” OR “story” OR “stories” OR “storytelling” OR “creative writing” OR “novel*” OR (narrative* W/5 (method* OR work* OR form* OR practice* OR art* OR creativ* OR engag* OR interact* OR output* OR outreach* OR display* OR disseminat* OR communicat*)) OR “installation*” OR “sculpture*” OR “2D” OR “two dimensional” OR “two-dimensional” OR “3D” OR “three dimensional” OR “three-dimensional” OR “poster*” OR “audiovisual*” OR “audio-visual*” OR “cinematograph*” OR “artifact*” OR “creation*”)) AND ((disseminat* W/5 (result* OR findings OR scien* OR knowledge OR research OR channel* OR tool* OR instrument* OR method* OR technique* OR strateg* OR practice*)) OR (“knowledge translation” OR (translat* W/5 (result* OR findings OR scien* OR knowledge OR research OR tool* OR instrument* OR method* OR technique* OR strateg* OR practice*))) OR (communicat* W/5 (result* OR findings OR scien* OR knowledge OR research OR channel* OR tool* OR instrument* OR method* OR technique* OR strateg* OR practice*)) OR (“output*” OR “outreach*” OR “publication*” OR “present*” OR “co-present*” OR “prototyp*” OR “display*” OR “represent*”))
**Terms to capture evaluation criteria:**
((“evaluation framework” OR “evaluation criteria” OR (evaluat* W/5 (criter* OR indicator* OR guide* OR marker* OR critical* OR quality OR impact OR perform* OR values OR credibility OR validity OR applicability OR framework OR instrument* OR tool* OR standard* OR dissemination OR communication OR translation))) OR (assess* W/5 (criter* OR indicator* OR guide* OR marker* OR critical* OR quality OR impact OR perform* OR values OR credibility OR validity OR applicability OR framework OR instrument* OR tool* OR standard* OR dissemination OR communication OR translation)) OR (“critical appraisal” OR (apprais* W/5 (criter* OR indicator* OR guide* OR marker* OR critical* OR quality OR impact OR perform* OR values OR credibility OR validity OR applicability OR framework OR instrument* OR tool* OR standard*))) OR (criter* W/5 (quality OR impact OR critical* OR perform* OR values OR credibility OR validity OR applicability OR dissemination OR communication OR translation)) OR (guide* W/5 (quality OR impact OR critical* OR perform* OR values OR credibility OR validity OR applicability OR dissemination OR communication OR translation)) OR (indicator* W/5 (quality OR impact OR critical* OR perform* OR values OR credibility OR validity OR applicability)) OR (marker* W/5 (quality OR impact OR critical* OR perform* OR values OR credibility OR validity OR applicability)))

### 2.2. Extraction and screening procedure

To determine the eligibility of the extracted studies retrieved through the initial search, we applied a set of inclusion and exclusion criteria based on four a priori premises: perspective, outcome, phenomenon of interest, and study domain. These premises, developed in accordance with the JBI guidelines, served as a conceptual framework to guide the screening process and ensured that included studies aligned with our objective: identifying evaluation criteria for creative and interactive public science dissemination.

1. *Perspective*: We included studies that evaluated the dissemination of scientific knowledge, i.e., knowledge generated through scientific methods. Science dissemination was considered a form of knowledge translation [3] that involves an intentional and active effort to make research findings accessible to various audiences [25].
2. *Outcome:* Studies were only included if they presented explicit criteria for evaluating the quality and/or impact of dissemination practices. We excluded studies that addressed communication or dissemination without any form of evaluative focus.
3. *Phenomenon of Interest:* We focused on evaluation criteria related to creative and/or interactive forms of science dissemination. Creative practices were defined as artistically inspired, imaginative, or sensorially rich ways of sharing research findings. Interactive practices referred to formats designed to involve citizens in a two-way exchange of information (e.g., quizzes, polls, or immersive experiences).
4. *Study domain:* We included studies from the social-behavioral sciences, educational sciences, and humanities, as well as interdisciplinary work involving these fields. Studies rooted solely in clinical or biomedical domains without a socio-educational component were excluded.

The retrieved studies were first collected in Zenodo and then transferred to an MS Excel file to support the screening rounds. The screening process consisted of three rounds. In the first round, the lead author removed duplicates and assessed the titles of the retrieved sources to ascertain their appropriateness for the review project’s purpose. Titles that were clearly off topic were excluded. Titles that were vague or incomprehensive were included.

In a second round, a team of twelve reviewers screened the abstracts of the remaining sources based on the inclusion criteria. Each reviewer received a list of abstracts, organized and tabulated in separate standard MS Excel sheets. Each abstract was independently assessed by two reviewers. An abstract was included if all four inclusion criteria were met and excluded if one or more of the criteria were not met. When in doubt, a reviewer could mark the abstract as ‘unclear’. In cases where abstracts were labelled as unclear or if there was a disagreement regarding inclusion (i.e., one reviewer let it pass for inclusion while the other did not), a third screening round was started to resolve the conflict. In this third screening round, a third reviewer screened the respective abstract and took a final decision regarding inclusion or exclusion of the study.

### 2.3. Synthesis

The findings were synthesized using a thematic synthesis approach, following the method described by Thomas and Harden (2008) [26]. This approach, commonly applied in qualitative research and systematic reviews, involves systematically coding and organizing data into descriptive and analytical themes [27]. In this review, the data that were charted—namely, the evaluation criteria extracted from the included studies—were first coded and categorized according to thematic similarity. During a first round, the first author of the study clustered the criteria, derived from the included studies, into two groups on an online Miro-board. One group contained broad criteria codes linked to themes as scientific content, creative design, interactivity, and impact of the science communication. The second group contained more narrow criteria codes applied to specialized themes such as game-based learning, complexity translation, transmedia, and communication via user-friendly technology. These codes were initially created through open coding, based on recurring terminology and concepts across the extracted data. In a second round, the three authors relabeled, rearranged and clustered the criteria codes using an online interactive Miro-board. This collaborative phase allowed for cross-validation and refinement of the identified themes.

### 2.4. Expert consultation: Face validity checks

The synthesis resulted in a first version of the evaluation framework with quality criteria. As a supplementary step to the scoping review, eight face validity checks were conducted between October 2021 and January 2024 through workshops and educational activities involving practitioners and academics. Face validity is a form of qualitative assessment in which experts or potential users determine whether an instrument or tool serves its intended purpose. In this case, the goal was to assess whether the framework is comprehensive and effectively supports the evaluation of science dissemination practices.

We conducted five face validity checks with practitioners in online interactive workshops using Miro; each workshop lasted about two hours. Participating practitioners represented media professionals, Human-Computer Interaction specialists, art educators, and museum professionals, and came from various geographical locations, namely Belgium, Finland, Egypt, Denmark, Sweden, Estonia, Malta, and the United Kingdom. As for the face validity checks within academia, we conducted two in person interactive sessions in Belgium that each lasted about two hours with bachelor and master level students. One session took place at the KU Leuven in the context of a Dutch bachelor course in social sciences and one at the University of Antwerp in the context of an international and intercultural postgraduate course “Asia – Europe Cultural Curatorship Studies”. The face validity process and results have been documented by the first author [4, p. 339, 28].

The participants of the online workshops and interactive sessions were invited to reflect on the use of the criteria in the light of their own science dissemination practices. They were also invited to report on their understanding of and experiences with the framework. This allowed us to qualitatively assess whether they understood what the criteria stand for, whether the content of these criteria was relevant and useful to make sense of their own science dissemination. Additionally, it allowed us to better understand how they experienced the framework as a whole, identify whether they had suggestions for adjustments to the framework, what criteria in the framework they had not considered before, and whether they thought there was something missing. The main results of the workshops and interactive sessions have been synthesized by the first author and documented in a report [28]. These face validity checks formed the basis for framework iterations and development. The comments by the academic and professional participants were used to inform both the content and the presentation of our review results.

After these eight face validity checks, the evaluation framework was revised and finalized. An additional face validity check of the final framework was conducted in January 2025 with an expert in science communication evaluation to ensure the framework was comprehensive.

## 3. Results

**Fig 1:**
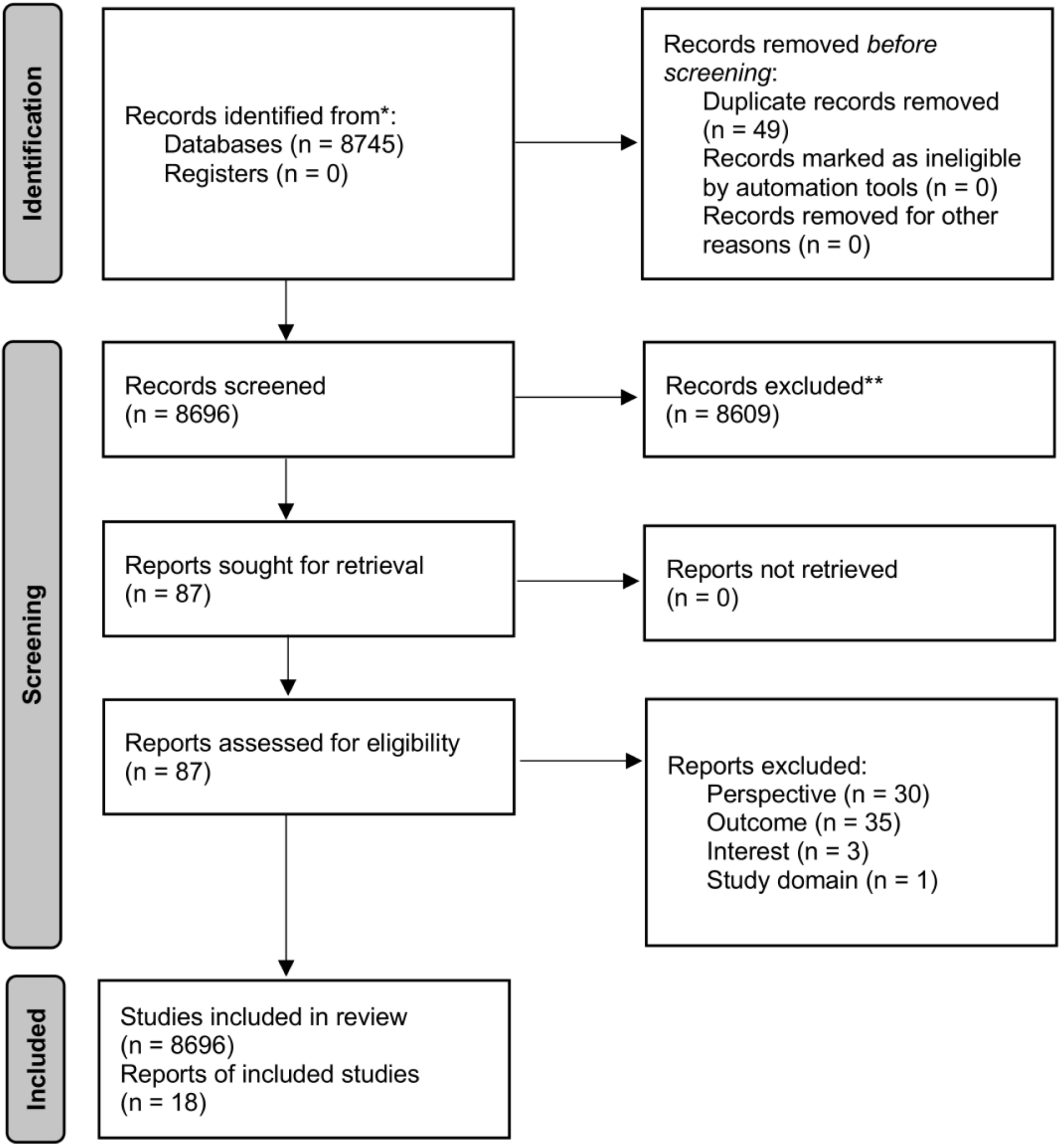
Overview of the screening process.

The search strategy identified 8745 studies initially. These studies underwent a screening procedure based on the inclusion criteria to determine their eligibility for the review. Duplicates were removed (n = 49) by the first author during the identification round, resulting in 8696 studies (Fig 1). During the first round of title screening by the first author, titles that were clearly off topic were removed (n = 2706). In a second and additional third round of abstract screening conducted by multiple independent reviewers, another 5903 studies were excluded, and 87 studies were deemed eligible for inclusion in the review. After the first author reviewed and analyzed the full text of the 87 studies based on the eligibility criteria, only 18 studies met all the inclusion criteria and were included in the final selection.

The first author created an MS Excel document with separate tabs for each of the 18 included studies, with each tab containing an in-depth analysis and an overview of the extracted criteria. Additionally, the same document included a tab listing the 69 studies that did not meet the criteria, along with justifications for their exclusion. The main reasons for exclusion at the full-text stage were: wrong perspective (n = 30), wrong outcome (n = 35), wrong interest (n = 3), and wrong study domain (n = 1).

The final list of 18 studies that met all four inclusion criteria span a diverse range of disciplines, methodologies, and thematic focus, reflecting the interdisciplinary and diverse nature of research on creative and interactive science dissemination. Several studies addressed the assessment of arts-based and aesthetic forms of knowledge translation. Some studies contributed design heuristics and frameworks for evaluating visual and multimodal science outreach. A number of studies focused on educational tools and game-based learning environments, offering evaluation dimensions related to usability, interactivity, and learning outcomes. Additionally, there were also studies that emphasized the importance of user-centered and human-centered design in the development of trustworthy and accessible science outreach, particularly for younger or lay audiences. The results also included studies that explored complexity in scientific storytelling, climate communication, and the integration of transmedia formats and immersive technologies. Furthermore, several studies proposed criteria related to technical quality, ethical considerations, and audience engagement. A concise list of the 18 included studies is provided in Table 2. Further details on the characteristics of the studies, as well as an overview of the analysis and synthesis, are available via OSF [23].

**Table 2:** Overview of the included studies.

Bibliography Reference	Studies
[29]	Lafrenière D, Cox SM. If you can call it a poem: Toward a framework for the assessment of arts-based works. Qualitative Research. 2013;13(3): 318–336. https://doi.org/10.1177/1468794112446104
[30]	Giang C, Piatti A, Mondada F. Heuristics for the development and evaluation of educational robotics systems. IEEE Transactions on Education. 2019;62(4): 278–287.
[31]	Polman JL, Gebre EH. Towards critical appraisal of infographics as scientific inscriptions. Journal of Research in Science Teaching. 2015;52(6): 868–893.
[32]	Piercy FP, Benson K. Aesthetic forms of data representation in qualitative family therapy research. Journal of Marital and Family Therapy. 2005; 31(1): 107–119.
[33]	Wirth V, Prutsch A, Grothmann T. Communicating climate change adaptation state of the art and lessons learned from ten OECD Countries. GAIA. 2014; 23(1): 30–39.
[34]	Wernbacher T, Wagner M, Rusch D, Hofsstaetter J. Learn by playing. Proceedings of the Vienna Games Conference. 2011: 775–777.
[35]	Nsangi, A, Semakula D, Rosenbaum SE, Oxman AD, Oxman M, Morelli A, et al. Development of the informed health choices resources in four countries to teach primary school children to assess claims about treatment effects: A qualitative study employing a user-centered approach. Pilot and Feasibility Studies. 2020;6(18):1–15.
[36]	Semakula D, Nsangi A, Oxman M, Rosenbaum SE, Oxman AD, Austvoll-Dahlgren A, et al. Development of mass media resources to improve the ability of parents of primary school children in Uganda to assess the trustworthiness of claims about the effects of treatments: A human-centred design approach. Pilot and Feasibility Studies. 2019;5(155). https://doi.org/10.1186/s40814-019-0540-4.
[37]	Newell R, Dale A, Winters C. A picture is worth a thousand data points: Exploring visualizations as tools for connecting the public to climate change research. Cogent Social Sciences. 2016; 2(1). https://doi.org/10.1080/23311886.2016.1201885.
[38]	Green DN, Du Puis JL, Xepoleas LM, Hesselbein C, Greder K, Pietsch V, et al. Fashion exhibitions as scholarship: Evaluation criteria for peer review. Clothing and Textiles Research Journal. 2019;39(1): 71–86.
[39]	Vervoort JM, Keuskamp DH, Kok K, van Lammeren R, Stolk T, Veldkamp TA, et al. A sense of change: Media designers and artists communicating about complexity in social-ecological systems. Ecology and Society. 2014;19(3). https://doi.org/10.5751/ES-06613-190310.
[40]	Trigano PC, Pacurar-Giacomini E. Toward a web-based environment for evaluation and design of pedagogical hypermedia. Educational Technology & Society. 2004; 7(3): 21–37.
[41]	Perry MS. Multimodal engagement through a transmedia storytelling project for undergraduate students. GEMA Online® Journal of Language Studies. 2020; 20(3): 19–40.
[42]	Sylaiou S, Mania K, Paliokas I, Pujol-Tost L, Killintzis V, Liarokapis F. Exploring the educational impact of diverse technologies in online virtual museums. International Journal of Arts and Technology. 2016;10(1): 54–84.
[43]	Tahir R, Wang AI. State of the art in Game Based Learning: Dimensions for Evaluating Educational Games. 11th European Conference on Game Based Learning. 2017: 641–650.
[44]	Hainey T, Connolly T, Boyle L. A refined evaluation framework for games-based learning. Proceedings of the 4th European Conference on Games Based Learning. 2011: 97–105.
[45]	Giannakos MN. The Evaluation of an e-Learning Web-based Platform. Proceedings of the 2nd International Conference on Computer Supported Education. 2010: 433–438.
[46]	Kukkonen T, Cooper A. An arts-based knowledge translation (ABTK) planning framework for researchers. Evidence & Policy. 2017; 15(2): 293–311.

Based on our thematic synthesis approach, we identified criteria codes that represented the original ideas derived from the retrieved studies. The criteria codes were reordered along the axes of three core themes: ‘scientific base’, ‘translation, presentation, and design’, and ‘impact’. Our analysis revealed that these three themes resonate well with the ‘normative’ (scientific base), ‘substantive’ (translation, presentation, and design) and ‘performative’ (impact and attitudes) layers of criteria as described by Lafrenière & Cox (2013) [29], a study included in the set of retrieved studies in our review. Therefore, we integrated their conceptual lens with our themes in the development of our evaluation framework. Each theme was further divided into categories and subcategories of criteria. This resulted in an overall evaluation framework presenting a taxonomy of criteria designed for evaluating the quality of creative and interactive dissemination practices and tools.

The face validity checks of the evaluation framework revealed that these themes and their categories were effective for the participants. No additional criteria emerged from the workshops; however, participants—both academic and professional—suggested clustering similar criteria under broader umbrella terms to make the framework more usable [4,28]. To address this, the initial version of the framework, which consisted of 153 criteria based on thematic codes, was refined to 125 criteria, as presented in the result section. The original version of the full framework remains accessible via OSF [23].

Additionally, most face validity participants indicated that they found it difficult to operationalize the criteria. To address this, we supplemented each of the 125 criteria by guiding questions. These questions would serve the dual purpose of elucidating the criteria’s meanings and supporting users in translating the abstract criteria into practical applications.

The final list of criteria and their guiding questions should not be treated as a checklist with simple ‘yes’ or ‘no’ answers. Instead, they are intended to initiate reflection. For example, if one reads the question “Have you considered using audio formats?”, one needs to consider whether an audio format is appropriate for the specific dissemination that is being reflected upon. In case there would be added value additional reflection questions need to be posed by the framework user such as “Which audio format would be suitable?” and “How will it interact with other sensorial components?”.

In what follows, we present the final evaluation framework, organized around three overarching themes that cluster ‘normative’, ‘substantive’ and ‘performative’ criteria. Each criterion is accompanied by a reference number corresponding to the bibliographic reference(s) from which it was derived.

### 3.1. Normative criteria

Normative criteria refer to the foundational scientific and ethical dimensions that underpin the integrity, rigor, and accountability of a science dissemination effort. They allow for the assessment of how well a dissemination practice adheres to scientific standards (e.g., methodological transparency, credibility, contextualization), and how ethically sound and socially responsible it is in representing knowledge, people, and values. This includes considerations such as research design, positionality, reliability, data accuracy, transparency, and representation of voices. These criteria help ensure that science dissemination does not compromise scientific quality or ethical principles in pursuit of accessibility or creativity.

**Table:** 

Normative criteria [29]: Scientific Base of the Science Dissemination		
Subcategory	Codes	Questions
*Category 1: Methodological criteria* [29,38]		
Relevance, purpose [30,31]	Rationale [31]	What is the underlying rationale behind the science dissemination?
	Meaning and value [31,32]	Does the science dissemination address issues of significance for society or the scientific community? Does it contribute to important issues?
	Appropriateness [29,31]	Is the science dissemination appropriate in the context of presentation? Is it befitting?
Scientific soundness and data (re)presentation [31,33]	Context [31,32,34]	Is the data presented with sufficient context to enable understanding and interpretation?
	Scale, magnitude [30,31]	Is the research presented in a balanced, proportional manner that avoids overgeneralization or exaggeration?
	Rigor [29,31–33,35,36]	Are the sources credible, and is the research transferable or replicable under similar circumstances?
	Completeness, sufficiency [31,32]	Was sufficient data collected and analyzed? Is cherry-picking avoided?
	Clarity [29,31,37,39]	Is the data clear and understandable? Is the data presented in a clear and comprehensible manner?
	Accuracy [31]	Is the scientific information depicted accurately and free of errors or misrepresentation?
	Scientific control [29,38]	Has the data been peer-reviewed, triangulated, or validated through rigorous scientific processes?
	Transparency [29,30,32,36,38,39]	Is the research process transparent? Can the audience access the sources, understand the funding origins, and recognize partnerships involved?
	Background researcher, positionality [32,38]	Do researchers openly disclose their positionality, biases, or influence on the work?
	Primary sources [33]	Does the dissemination rely on accurate, verified primary data sources?
	Lived experience [33,38]	Does the dissemination include and authentically represent the lived experiences of individuals or groups it references?
	Trustworthiness, reliability, robustness [29,32,33,35]	Are the procedures reliable and the interpretations robust? Is the content trustworthy and aligned with accepted scientific standards?
*Category 2: Ethical criteria *[29]		
Representation of people	Represented voices [38]	Are the perspectives and voices of those represented in the dissemination authentically included?
	Anonymity [29]	Are the identities of individuals protected where necessary? If identities are disclosed, is this done with their consent?
Integrity principles [29,32]	Authorship, contributions [29]	Are all contributors properly credited for their work and contributions?
	Harms and benefits [29]	Does the science dissemination aim to benefit society, and does it avoid causing harm?
	Sensitivity [32]	Is the content presented in a way that respects the sensitivities of the topic, individuals, or communities involved?
	Accountability [32]	Do the science communicators take responsibility for the potential societal impact of their communication?
	Respectful [32]	Is the tone and presentation of the science dissemination respectful to all stakeholders and audiences?

### 3.2. Substantive criteria

Substantive criteria concern the way scientific content is translated, designed, and presented through various media and formats, and how this shapes its accessibility, clarity, and meaning for public audiences. This includes the structuring and aesthetic framing of content, the use of multimodal communication (visual, audio, textual, gestural, etc.), and the integration of complexity or uncertainty in the representation of science. These criteria also assess the alignment between the content and its form, the intentionality behind design decisions, and the extent to which the dissemination is understandable, engaging, and theoretically grounded. Substantive criteria emphasize the communicative craft of science dissemination. This wide range of criteria was structured under three categories, comprising content translation and presentation criteria, design criteria, and interactive tools criteria.

**Table:** 

Substantive criteria [29]: Translation, Presentation, and Design of the Science Dissemination		
Subcategory	Codes	Questions
*Category 1: Content translation and presentation criteria*		
Reach, resonance [38]		Does the dissemination resonate with a broad and diverse audience?
Theoretical engagement [38]		Is there a clear theoretical foundation (explicit or implicit) for the dissemination or display? How has theory been applied, implemented, or generated from the research and presentation process? Alternatively, how has new theory emerged from the research process and presentation?
Assessment [29]		Is the science dissemination critically evaluated before, during, and after the presentation to ensure effectiveness?
Retrievability [37,38]		Will the content or outcome be accessible after the presentation concludes? How can it be retrieved?
Display order, organization [31]	Organization of information [31,39,40]	How is the information structured, grouped, or organized?
	Critical evaluation [38]	Is the sequence of displayed information critically and thoughtfully constructed?
	Intentional [31]	Is the order of the information intentional and logical?
Evidence [38]	Ethical approach [38]	Is there evidence of ethical considerations in selecting the presented information?
	Scientific dimensions [38]	Is the dissemination scientifically grounded and is the scientific dimension well-integrated in the communication?
	Display process [38]	Is the process of selecting and displaying information clarified? Are relationships between elements clarified?
Complexity translation [37,39]	Feedback [39]	Does the dissemination include feedback loops or demonstrate interconnectedness?
	Uncertainty [39]	Does it reflect the iterative and plausible nature of science?
	Nonlinearity [39]	Does the dissemination avoid linear storytelling, integrating diverse elements into a cohesive whole?
	Path-dependency [39]	is the path-dependence (positionality) transparent in the dissemination? Is the influence of previous choices transparent in the dissemination?
	Openness, multiple perspectives [32,39]	Does the dissemination allow for multiple interpretations or perspectives?
	Scale dynamics [37,39]	Is the representation dynamic and reflective of change or fluidity?
Modes of meaning [41]	Visual [40,41]	Have you considered using visual formats such as didactical pictures, photography, drawing, animation, images,…? Do you thoughtfully consider and apply visual elements such as color, perception,…?
	Textual and linguistic [31,40,41]	Have you considered using text formats such as writing, speech, dialogue, redaction, typography,..? Is page design well-thought-out?
	Gestural, kinetic, embodied, haptic [41]	Have you considered incorporating physical or gestural modes such as body language?
	Sound, audio [40,41]	Have you considered using audio formats such as speech, music, sound effects,…?
	Technical, digital [41]	Have you considered using technical modes of meaning? Do you consider camera angle, distance, brightness,…? Are technical aspects such as camera angles, brightness, and digital enhancements used in a meaningful manner?
	Interdependency [40]	Have you considered how multiple modes of meaning interact, complement, or compete with one another?
*Category 2: Design criteria *[38]		
Appropriate form [33,38]		Is the chosen format, medium, or channel suitable for conveying the intended information?
Cohesiveness, alignment [31,38]		Is there conceptual alignment across the design elements? Is the information well-integrated in the design? Is there cohesiveness from a conceptual perspective?
Vividness [37]		Does the outcome leave a lasting impression or stand out vividly?
Message translation [31]		Is the intended message clearly conveyed through the design? Were choices in the design translation process deliberate and meaningful?
Space [31,38]	Accessibility [38]	Is the presentation space inclusive and accessible to individuals with disabilities or those unable to attend in person?
	Virtual spatiality [42]	Is there a (virtual) extension to the physical presentation space?
	Effectiveness [31]	Is the space utilized effectively for the dissemination?
Iterative revisions and reflection [38]	Outcome [38]	Were iterative processes documented, and can they offer insights for others?
	Original concept [38]	How has the original concept evolved during the process, and is it still connected to the final outcome?
	Internal reflection [38]	Was the presentation evaluated and revised internally, with openness to critique?
Aesthetic merit [32,37]	Attractiveness [37]	Is the design appealing?
	Aesthetic standards [32]	Does it meet accepted artistic or design standards? Does it meet the standards of good art, writing, drama,…?
	Data and art spectrum [37]	Does it strike a balance between artistic expression and accurate data representation?
Facilitating understanding [31]	Good example [33]	Does the dissemination relate to good examples? Does the design translation contain a good example?
	Avoidance of distractions [37]	Are there elements that may distract people instead of support them? Are distracting elements minimized?
	Clarity and readability [31,33,37,39]	Are concepts clearly defined and easily understood?
	Understandability [31,35–37]	Can the audience understand the content? Is the text readable?
	Effectiveness communication and display [31,33,37]	Is the layout effective in visually and cognitively presenting the data?
*Category 3: Interactive tool criteria*		
Personalization, relatability, and interactive features of the tool [30,35–37,40,42]	Audience engagement, involvement [32,39,42,43]	Does the tool foster audience involvement and participation?
	Communication and collaboration [30,37,42]	Does the tool encourage communication and interaction among users? Is there an element of interpersonal interactivity?
	Familiarity [35,36]	Does the design of the tool feel relatable and familiar to users?
	Appropriateness [36]	Are the design elements contextually appropriate for the audience? Is the terminology appropriate? Are the examples or claims, stories and music genres appropriate for the target audience?
	Local knowledge [33]	Do the interactive features actively incorporate local knowledge or cultural identity?
	Local identity [33]	Do the interactive features reflect and engage with local identity?
	Elicit emotions [33]	Does the interactive experience evoke emotions in users? If so, is this done ethically and respectfully?
	Existing norms and values [33]	Do the interactive features align with and reflect existing social norms and values?
	Everyday life [33]	Do the interactive features connect to or translate meaningfully into users’ daily lives?
	Audience preferences [31,33,44]	Are the interactive features designed to align with the preferences of the targeted audience?
	Audience perspectives [31]	Do the interactive elements actively include and represent the perspectives of the audience?
Story support	Design mechanisms [43]	Do the tool’s design features support the storytelling process?
	Narration [40,42]	Is the narrative compelling and engaging?
	Visualizations [31,33]	Are visuals effectively employed to enhance storytelling?
Attractiveness, motivation [33,34,43,44]	Desirability [35,36]	Does the tool capture and maintain attention with novel or surprising elements? Does it attract and keep the attention of the audience?
	Aesthetics [30]	Is the tool visually appealing?
	Enjoyment [30,43]	Will users find the tool enjoyable and satisfying?
Playability [34,43]	Immersion [43]	Does the tool provide an immersive experience?
	Fun of play [34]	Is it fun to use?
Technical quality, realization [31,42]	Imageability, perceptual quality [31,42]	Does the tool have strong visual and perceptual quality?
	Sound [34]	Is the sound high quality?
	Graphics [34]	Are the graphics high quality?
	Controls [34]	Are controls intuitive and functional?
	Feasibility [36]	Is the tool practical and affordable to implement?
	Self-reliant [36]	Can the tool be used without constant assistance?
	Navigability [40,42]	Is the tool easy to navigate and use?
User-friendly tool, usefulness, and usability of the tool [34–36,40,43–45]	Practical value [35]	Is the tool practical?
	Effectiveness and efficiency [45]	Is the tool tested for user experience effectiveness and efficiency?
	Easy and satisfying [35,45]	Is the tool straightforward and satisfying to use?
	Consistency interface [40,45]	Is the interface consistent throughout?
	Guidance, efficient support, manuals [40,43,45]	Are manuals or support systems provided for user assistance? Is there support for the user?
	User manipulation, proactivity [40,45]	Does the tool allow users to make decisions and engage actively?
	Adaptability, flexibility [30,36,40]	Can the tool flexibly adapt to varied user needs?
	Level of automation [30]	Is the tool appropriately automated to meet user needs?
	Clear error messages [45]	Are error messages clear and helpful?
	Exit [45]	Is there an easy and clear way to exit the tool?
	Avoid unnecessary elements [39,45]	Is the tool minimalistic? Do all elements have a function? Are there distractive elements? Are all elements supportive? Does it convey the right amount of information? Does the tool avoid unnecessary distractions and present only essential elements?
	Comfort op physical setup [30]	Is the physical or digital setup comfortable for users?
	Shortcuts [45]	Are there shortcuts in its use?
	Minimalize cognitive workload [30,40,45]	Does the tool minimize cognitive demands?
	Level of difficulty [30,34,40,44]	Is the tool adjusted to the learning environment or situation?
	Comprehensive language [36,45]	Are instructions clear and comprehensible?
	Intuitive design [39]	Can users operate the tool easily without extensive instructions?

### 3.3. Performative criteria

Performative criteria address the effects and experiences generated by a science dissemination practice. They assess how the audience responds to and interacts with the dissemination in terms of engagement, emotions, reflection, and understanding. These criteria also consider longer-term impacts, such as knowledge gain, behavior change, policy influence, or collaborative outcomes. We distinguish between affect-related criteria (how dissemination is felt and experienced, e.g., emotional response, reflective attitudes) and effect-related criteria (what the dissemination achieves or produces, e.g., learning outcomes, social impact). These criteria help assess the transformative and participatory potential of science dissemination.

**Table:** 

Performative criteria [29]: Impact of the Science Dissemination		
Subcategory	Code	Questions
*Category 1: Affect criteria*		
Attitudes	Attitudes towards design and tools [44]	How did people react to the design and the tools used in the science dissemination? Where they affected by their interaction with these tools?
	Reflective attitudes towards taught subject [44]	Does the science dissemination encourage reflection on the subject matter? Does it facilitate deeper thinking or insights?
Emotions, feeling [29,35,40]	Reassuring – disconcerting [40]	Does the audience perceive the science dissemination as reassuring or disconcerting? What emotions does it evoke?
	Playful – serious [40]	Do the participants engage with the science dissemination in a playful or serious manner?
	Active – passive [40]	Is the audience active or passive when interacting with the science dissemination? Are they engaged or more passive in their interaction?
*Category 2: Effect criteria (12)*		
Reach [46]		How many people were reached? How many stakeholders were involved?
Awareness [46]		Does the experience encourage critical thinking? Does it foster awareness and consciousness about the subject matter?
Accessibility [29,39,40,46]		Was the science dissemination accessible to a wide range of people, including those with different abilities? Were the tools and design accessible to all?
Engagement, involvement [32,46]		Was the audience actively engaged with the content? How involved were the participants in the dissemination process?
Fascination, interest [31,34]		Did the science dissemination maintain the interest of the audience? Were they captivated or fascinated by the content?
Response, debate, dialogue [29,46]		Did the science dissemination lead to responses, debates, or active dialogue among the audience? Was there active interaction?
Partnership, collaboration [44,46]		Was there collaboration or co-production in the dissemination process with partners or target audiences?
Change [29]	Commitments [46]	Are there measurable commitments to change as a result of the dissemination? How are process and outcome measures tracked?
	Constructive action [32]	Did the dissemination lead to constructive actions or decisions? Did it inspire participants to take positive steps?
	Policy and advocacy [46]	Did the science dissemination influence policy debates, formation, or implementation? Were changes brought about through the dissemination process?
Usefulness [46]	Understanding [29,31,32]	Did the audience develop a clear understanding of the content, purpose, or message of the dissemination?
	Knowledge [46]	Did the audience gain knowledge from the dissemination? Did they intend to use the information presented or adapt it to their needs?
	Social phenomenon [32]	Did the audience understand the social phenomenon underlying the science dissemination? Did it provide new insights into the broader societal context?
New knowledge [38]	The field [38]	Did the science dissemination contribute new knowledge to the scientific or artistic field?
	Concept [38]	Was the concept behind the science dissemination novel or innovative?

## 4. Discussion

This study set out to identify and map criteria to develop a conceptual framework to assess the quality of interactive and creative public science dissemination. The findings of the scoping review showed that the quality criteria relevant to assessing the value of scientific knowledge translation and transfer could be clustered along three overarching themes: normative criteria (addressing the scientific base of the dissemination), substantive criteria (covering translation, presentation, and design of the science dissemination), and performative criteria (focusing on the impact of the dissemination). Each theme could be further divided into categories. More particularly, the normative criteria theme was divided into two categories: methodological criteria and ethical criteria. The substantive criteria theme comprised three categories: content translation and presentation criteria, design criteria, and interactive tool criteria. Finally, the performative criteria theme consisted of two categories: affect criteria and effect criteria.

Our framework differs from previously developed frameworks for the quality assessment of science communication, such as those by Lafrenière & Cox (2013) [29] and Olesk et al. (2021) [20], in various respects. First, our framework was not intended as a model to specifically assess the quality and effectiveness of the use of art in research dissemination, whereas the framework by Lafrenière & Cox (2013) focused solely on safeguarding artistic and scientific merit in arts-based creations [29]. Our framework is broader, aiming to develop an overall evaluation guide for science dissemination to support and improve public outreach efforts by identifying criteria relevant to a wide range of creative and interactive dissemination practices. While the use of art was included as one form of creative and interactive science dissemination in our review, it was not our specific focus. For example, digital media were also considered as enabling creative and interactive dissemination without involving art. Consequently, our framework was complemented by studies reporting on other lenses, approaches, and goals, such as the study of Vervoort et al. (2014) [39] introducing the issue of complexity in scientific knowledge translation and dissemination, and the study of Wernbacher et al. (2011) [34], reporting on the user-friendliness and usability of interactive tools. We further added nuance to the performative theme by subdividing it into criteria that assess both the effects and affects of dissemination practices.

Second, our study also differs from previous work on the quality assessment of science communication which tended to be more generic and did not focus specifically on creative and/or interactive dissemination practices. For instance, Olesk et al. (2021) developed a more generic framework of quality indicators for science communication. Their framework, based on six expert workshops, identified twelve valuable quality indicators, clustered into three themes: trustworthiness and scientific rigor, presentation and style, and connection with the society [20]. Although their focus was not on creative and/or interactive dissemination practices, the twelve indicators they identified were also present in the literature we analyzed and have been integrated into our own framework. This overlap demonstrates the robustness and broad applicability of our framework. Moreover, their framework shared a similar view on the purpose of assessment, recommending the use of criteria (or indicators) as reflective instruments for communicators rather than as normative tools for external judgement.

Third, our framework incorporates several criteria not previously reported, Including the consideration of multiple modes of meaning and their interdependency, the technical quality of communication tools, and reflective attitudes toward the subject and the design. These additions enhance the framework’s nuance and level of detail.

Our framework has various strengths. By incorporating various themes and providing an overview of detailed criteria, it offers a broad yet detailed foundation for assessing public science dissemination efforts. It functions as a tool to encourage interactive and/or creative science dissemination practices, supporting reflective and effective public science outreach. By synthesizing the literature, we integrated different lenses, approaches, and research fields, addressing the fragmentation of relevant research and bringing them together into a single framework. To make these evidence-based criteria actionable, we defined guiding questions to spark reflection and operationalize criteria for its uptake by science communicators. Additionally, we see potential in using these criteria not merely for summative evaluation, but also when planning, designing, and implementing interactive and creative science dissemination.

The current study does, however, also present specific challenges and inherent limitations. First, one of the limitations of the framework lies in the potential for its misapplication or misuse. The framework is intended to be used as a cohesive whole, with criteria from its three thematic sets – normative, substantive, and performative – considered in conjunction. However, in practice, science communicators may be tempted to disproportionately use substantive or performative criteria, particularly focusing on audience engagement or emotional impact, while overlooking the normative foundation, which relates to scientific soundness and ethics. Such fragmented use or imbalance could lead to dissemination delivered in the (authoritative) name of science, that is appealing but lacking in robustness, and therefore at risk of becoming misleading or even propagandistic [4, p. 159]. In such cases, an ‘entertaining’ format may overshadow the underlying evidence, potentially misleading audiences and undermining trust in science communication.

The responsibility for any such misuse ultimately rests with the users of the framework and their commitment to upholding scientific integrity. Although we cannot control how the framework will be applied, we emphasize that its value lies in supporting a responsible and reflective approach to science dissemination. A balanced application that draws on all three sets of criteria ensures that dissemination efforts are not only engaging and accessible, but also grounded in trustworthy science and ethical practice.

Second, we view the findings of the current study not as a conclusion, but as an intermediate step towards further research, knowledge translation, and tool development. For instance, there is an implicit assumption that professional science communicators will be interested in adopting these criteria as guiding principles for evaluating, and potentially even proactively designing, science dissemination. Nevertheless, a significant challenge lies in encouraging practitioners to engage with the framework in real-world settings. The complexity of the terminology and the high number of criteria might complicate its uptake and discourage widespread use.

As the framework is comprehensive and contains many subcategories, it remains difficult to apply without sufficient support. While reducing the number of criteria was an effort to enhance usability, we acknowledge that the framework may still feel overwhelming to some users. Making the framework more user-friendly requires rethinking its format, a step that could come at the expense of nuance and detail. However, since this study is a scoping review, our *aim* was to gather an exhaustive set of evaluation criteria, previously scattered across the literature, into one coherent and sound overview that can now serve as a foundation for evidence-based future tool development.

To support implementation and discourage superficial or instrumental use, such as treating the framework as a box-ticking checklist, we have aimed to operationalize the criteria by linking each one to a reflective guiding question. These prompts help practitioners apply the criteria within their specific-context and critically assess whether their communication is grounded in scientific rigor (normative), clearly and accessibly presented (substantive), and meaningfully engaging to audiences (performative).

Still, we recognize that reflective questioning alone may not provide sufficient clarity on what constitutes a “successful” application of the framework. Our ongoing research will therefore explore the practical use of the framework in applied settings to assess whether users can meaningfully prioritize or weight criteria depending on their specific contexts. In addition, further development focuses on presenting the evaluation framework in a more creative and interactive format, such as our card-based reflection tool, available on Zenodo [47], to better facilitate its integration into real-world science communication practices.

Third, the framework consists of a set of quality criteria. The term ‘criteria’ refers to principles or standards of judgement, while the term ‘quality’ denotes distinctive characteristics, attributes, or conditions used as benchmarks for comparing similar things, often implying a degree of excellence by those that use the term ‘quality’. This raises important questions: who determines what constitutes ‘excellence’ in science communication, whose practices are considered to be ‘excellent’, and what or who may have been overlooked in such comparison [4, pp. 160–161]. The search phase of the review focused exclusively on information sources retrieved from scientific databases, written in English, and contained scientific terminology. As a result, while the included sources retrieved through the literature review process represented a wide variety of disciplines and professions, we acknowledged that they might lack a broad diversity of social and cultural perspectives regarding quality. We did identify studies authored by scholars from various cultural backgrounds within the retrieved English-language literature. However, recognizing that relying solely on English-language sources could limit the diversity of perspectives, we took additional steps to ensure the comprehensiveness of our mapping results. This limitation was further addressed through international face validity checks, covering geographical regions with no native English-speaking populations, including Belgium, Finland, Egypt, Denmark, Sweden, and Malta. These checks extended beyond the regular review process by engaging and consulting practitioners and academics from various countries with diverse social and cultural backgrounds.

Moreover, in the ongoing follow-up phase of the research project where the criteria were translated into reflective questions for an interactive tool [47], we have already included participants from diverse cultural and social backgrounds for additional face validity checks, covering geographical locations including China, Korea, Iran, Thailand, Finland, Belgium, India, Portugal, Nigeria, and Italy [forthcoming publication]. To date, no additional criteria have been identified during these international expert consultations. The face validity checks resulted mainly in (re)clustering existing criteria, operationalizing the framework, and enhancing its usability as a practical tool.

We did not find evidence to suggest that including non-English sources in our initial search would generate entirely new criteria, as demonstrated through multiple international participative workshops conducted over the past two years. This does not preclude, however, the potential for further research on cultural and social nuances, particularly in non-academic or grey literature.

## Conclusion

This study set out to examine what criteria are described and used in the literature to assess the quality and value of creative and interactive science dissemination for public outreach purposes. Through a scoping review of the literature, we identified and analyzed 18 studies that met our inclusion criteria (perspective, outcome, phenomenon of interest, study domain). From these, we developed a comprehensive evaluation framework structured around three overarching criteria themes. The first theme includes normative criteria, which address the scientific foundation and ethical considerations of science dissemination. The second theme considers substantive criteria, which focus on the translation, presentation, and design of science dissemination. The third theme refers to performative criteria, which relate to the impact of science dissemination on audiences. Each criterion identified within these themes was supplemented by reflective guiding questions to support operationalization, foster contextual application, and encourage critical application. The resulting framework not only consolidates previously scattered insights but also offers science communicators a tool for improving the quality and impact of their public outreach efforts.

The primary goal of this review was to enhance the value and quality of science dissemination by equipping science communicators with guidelines to assess dissemination practices for public outreach. By offering a comprehensive and interdisciplinary synthesis of the literature, this review contributes to the advancement of science outreach practices, ensuring they are engaging, inclusive, impactful, and scientifically grounded. Ultimately, this framework represents a step towards elevating the standards of science communication and strengthening the relationship between science and society in meaningful ways. While the framework has been primarily developed as a tool for evaluation, it can also act as a proactive guide for creating valuable public science dissemination initiatives. Additionally, it provides a foundation for evidence-based future tool development.

## Supporting information

S1 File PRISMA 2020 flow diagram. (DOCX)

S2 File PRISMA ScR checklist. (DOCX)

## References

[1] Scherer J, Weber S, Afzofra M, Ruete A, Sweeney E, Weiler N. Making the most of your H2020 project: Boosting the impact of your project through effective communication, dissemination and exploitation. European IP Helpdesk: European Union; 2019. doi: 10.2826/045684

[2] Edwards DJ. Dissemination of research results: on the path to practice change. Can J Hosp Pharm. 2015;68(6):465–9. doi: 10.4212/cjhp.v68i6.1503 26715783 PMC4690672

[3] Wilson PM, Petticrew M, Calnan MW, Nazareth I. Disseminating research findings: what should researchers do? A systematic scoping review of conceptual frameworks. Implement Sci. 2010;5:91. doi: 10.1186/1748-5908-5-91 21092164 PMC2994786

[4] Van Even P. Transcending the horizon of public science dissemination: A foundational philosophical reflection on the science communication paradigm. Leuven: KU Leuven; 2023. ISBN: 9789081428019

[5] Hecker S, Haklay M, Bowser A, Makuch Z, Vogel J, Bonn A. Citizen science: Innovation in open science, society and policy. London: UCL Press; 2018.

[6] Wynn J. Citizen science in the digital age: rhetoric, science, and public engagement. Alabama: The University of Alabama Press; 2017.

[7] Dearing JW, Kee KF. Historical roots of dissemination and implementation science. In: Brownson RC, Colditz GA, Proctor EK, editors. Dissemination and implementation research in health: Translating science to practice. New York: Oxford University Press; 2012. pp. 55–71.

[8] Rolling J. Arts-based research in education. In: Leavy P, editor. Handbook of arts-based research. New York: The Guilford Press; 2017. pp. 493–510.

[9] Barab S, Squire K. Design-based research: putting a stake in the ground. J Learn Sci. 2004;13(1):1–14. doi: 10.1207/s15327809jls1301_1

[10] Bailey NM, Van Harken EM. Visual images as tools of teacher inquiry. J Teach Educ. 2014;65(3):241–60. doi: 10.1177/0022487113519130

[11] McGregor C. Art-informed pedagogy: tools for social transformation. Int J Lifelong Educ. 2012;31(3):309–24. doi: 10.1080/02601370.2012.683612

[12] Sinner A. The visual journal as an image sphere: interpreting artworks with an anamorphic perspective. Stud Art Educ. 2011;52(3):183–95. doi: 10.1080/00393541.2011.11518834

[13] Jenkins A, Healey M. Undergraduate research and international initiatives to link teaching and research. Council Undergrad Res Q. 2010;30:36–42.

[14] Bazeley P. Research dissemination in creative arts, humanities and the social sciences. Higher Educ Res Dev. 2006;25(3):307–21. doi: 10.1080/07294360600793101

[15] Ward V, House A, Hamer S. Developing a framework for transferring knowledge into action: a thematic analysis of the literature. J Health Serv Res Policy. 2009;14(3):156–64. doi: 10.1258/jhsrp.2009.008120 19541874 PMC2933505

[16] Hailey D, Grimshaw J, Eccles M, Mitton C, Adair CE, McKenzie E, et al. Effective dissemination of findings from research. Edmonton: Institute of Health Economics; 2008.

[17] Sudsawad P. Knowledge translation: Introduction to models, strategies, and measures. Austin: The National Center for the Dissemination of Disability Research; 2007.

[18] Fischhoff B. The sciences of science communication. Proc Natl Acad Sci U S A. 2013;110 Suppl 3(Suppl 3):14033–9. doi: 10.1073/pnas.1213273110 23942125 PMC3752164

[19] Schäfer MS. Mediated trust in science: concept, measurement and perspectives for the `science of science communication’. J Sc Commun. 2016;15(05):C02. doi: 10.22323/2.15050302

[20] Olesk A, Renser B, Bell L, Fornetti A, Franks S, Mannino I, et al. Quality indicators for science communication: Results from a collaborative concept mapping exercise. J Sci Commun. 2021;20(3). doi: 10.22323/2.20030206

[21] Mannino I, Bell L, Costa E, Di Rosa M, Fornetti A, Franks S, et al. Supporting quality in science communication: insights from the QUEST project. J Sci Commun. 2021;20(3). doi: 10.22323/2.20030207

[22] Van Even P. Protocol for a systematic review of evaluation criteria for creative and interactive dissemination practices. Zenodo. 2022. Available from: https://zenodo.org/records/14397251

[23] Van EvenP. Quality criteria for evaluating creative and interactive science dissemination in public outreach efforts: A scoping review. OSF; 2025. doi: 10.17605/OSF.IO/A8YJ940705821

[24] Ortiz-Ospina E. The rise of social media. Our World in Data. 2019. Available from: https://ourworldindata.org/rise-of-social-media

[25] Rabin BA, Brownson RC, Haire-Joshu D, Kreuter MW, Weaver NL. A glossary for dissemination and implementation research in health. J Public Health Manag Pract. 2008;14(2):117–23. doi: 10.1097/01.PHH.0000311888.06252.bb 18287916

[26] Thomas J, Harden A. Methods for the thematic synthesis of qualitative research in systematic reviews. BMC Med Res Methodol. 2008;8:45. doi: 10.1186/1471-2288-8-45 18616818 PMC2478656

[27] Nicholson E, Murphy T, Larkin P, Normand C, Guerin S. Protocol for a thematic synthesis to identify key themes and messages from a palliative care research network. BMC Res Notes. 2016;9(1):478. doi: 10.1186/s13104-016-2282-1 27769317 PMC5073737

[28] Van Even P, Zaman B, Hannes K. ParCos Deliverable 3.4: Evaluation report on science communication guidelines. Lappeenranta: Finland; 2022.

[29] Lafrenière D, Cox SM. ‘If you can call it a poem’: toward a framework for the assessment of arts-based works. Qualit Res. 2012;13(3):318–36. doi: 10.1177/1468794112446104

[30] Giang C, Piatti A, Mondada F. Heuristics for the development and evaluation of educational robotics systems. IEEE Trans Educ. 2019;62(4):278–87. doi: 10.1109/te.2019.2912351

[31] Polman JL, Gebre EH. Towards critical appraisal of infographics as scientific inscriptions. J Res Sci Teach. 2015;52(6):868–93. doi: 10.1002/tea.21225

[32] Piercy FP, Benson K. Aesthetic forms of data representation in qualitative family therapy research. J Marital Fam Ther. 2005;31(1):107–19. doi: 10.1111/j.1752-0606.2005.tb01547.x 15739971

[33] Wirth V, Prutsch A, Grothmann T. Communicating climate change adaptation. State of the art and lessons learned from ten OECD countries. GAIA - Ecol Perspec Sci Soc. 2014;23(1):30–9. doi: 10.14512/gaia.23.1.9

[34] Wernbacher T, Wagner M, Rusch D, Hofsstaetter J. Learn by playing. Proceedings of the Vienna Games Conference. 2011. 775–7.

[35] Nsangi A, Semakula D, Rosenbaum SE, Oxman AD, Oxman M, Morelli A. Development of the informed health choices resources in four countries to teach primary school children to assess claims about treatment effects: a qualitative study employing a user-centered approach. Pilot Feasibility Stud. 2020;6(18):1–15. 32055405 10.1186/s40814-020-00565-6 PMC7008535

[36] Semakula D, Nsangi A, Oxman M, Rosenbaum SE, Oxman AD, Austvoll-Dahlgren A, et al. Development of mass media resources to improve the ability of parents of primary school children in Uganda to assess the trustworthiness of claims about the effects of treatments: a human-centred design approach. Pilot Feasibility Stud. 2019;5:155. doi: 10.1186/s40814-019-0540-4 31890267 PMC6935490

[37] Newell R, Dale A, Winters C. A picture is worth a thousand data points: Exploring visualizations as tools for connecting the public to climate change research. Cogent Soc Sci. 2016;2(1):1201885. doi: 10.1080/23311886.2016.1201885

[38] Green DN, Du Puis JL, Xepoleas LM, Hesselbein C, Greder K, Pietsch V, et al. Fashion exhibitions as scholarship: evaluation criteria for peer review. Cloth Textil Res J. 2019;39(1):71–86. doi: 10.1177/0887302x19888018

[39] Vervoort JM, Keuskamp DH, Kok K, van Lammeren R, Stolk T, Veldkamp TA, et al. A sense of change: media designers and artists communicating about complexity in social-ecological systems. Ecol Soc. 2014;19(3). doi: 10.5751/es-06613-190310

[40] Trigano PC, Pacurar-Giacomini E. Toward a web-based environment for evaluation and design of pedagogical hypermedia. Educ Technol Soc. 2004;7(3):21–37.

[41] Perry MS. Multimodal engagement through a transmedia storytelling project for undergraduate students. J Lang Stud. 2020;20(3):19–40. doi: 10.17576/gema-2020-2003-02

[42] Sylaiou S, Mania K, Paliokas I, Tost LP, Killintzis V, Liarokapis F. Exploring the educational impact of diverse technologies in online virtual museums. Int J Arts Technol. 2017;10(1):58. doi: 10.1504/ijart.2017.083907

[43] Tahir R, Wang AI. State of the art in game based learning: dimensions for evaluating educational games. 11th European Conference on Game Based Learning. 2017. pp. 641–50.

[44] Hainey T, Connolly T, Boyle L. A refined evaluation framework for games-based learning. Proceedings of the 4th European Conference on Games Based Learning. 2011. pp. 97–105.

[45] Giannakos MN. The evaluation of an e-learning web-based platform. Proceedings of the 2nd International Conference on Computer Supported Education. 2010. pp. 433–8.

[46] Kukkonen T, Cooper A. An arts-based knowledge translation (ABKT) planning framework for researchers. Evid Policy. 2019;15(2):293–311. doi: 10.1332/174426417x15006249072134

[47] Van Even P. Trainer Deck Cards. Zenodo; 2022. Available from: https://zenodo.org/records/7589281

