# Supplementary material for: Quality criteria for evaluating creative and interactive science dissemination in public outreach efforts: A scoping review
Source: PLoS One. 2025 Jul 24;20(7):e0328800. doi: 10.1371/journal.pone.0328800 (PMC12289055; doi:10.1371/journal.pone.0328800)
Supplement: S1 File — (DOCX) [file pone.0328800.s001.docx]

**Identification of studies via databases and registers**

Records removed *before screening*:

Duplicate records removed (n = 49)

Records marked as ineligible by automation tools (n = 0)

Records removed for other reasons (n = 0)

Records identified from*:

Databases (n = 8745)

Registers (n = 0)

**Identification**

Records screened

(n = 8696)

Records excluded**

(n = 8609)

Reports sought for retrieval

(n = 87)

Reports not retrieved

(n = 0)

**Screening**

Reports assessed for eligibility

(n = 87)

Reports excluded:

Perspective (n = 30)

Outcome (n = 35)

Interest (n = 3)

Study domain (n = 1)

Studies included in review

(n = 8696)

Reports of included studies

(n = 18)

**Included**

*Consider, if feasible to do so, reporting the number of records identified from each database or register searched (rather than the total number across all databases/registers).

**If automation tools were used, indicate how many records were excluded by a human and how many were excluded by automation tools.

Source: Page MJ, et al. BMJ 2021;372:n71. doi: 10.1136/bmj.n71.

This work is licensed under CC BY 4.0. To view a copy of this license, visit <https://creativecommons.org/licenses/by/4.0/>
